# Deployment of Spatial Attention towards Locations in Memory Representations. An EEG Study

**DOI:** 10.1371/journal.pone.0083856

**Published:** 2013-12-26

**Authors:** Marcin Leszczyński, Agnieszka Wykowska, Jairo Perez-Osorio, Hermann J. Müller

**Affiliations:** 1 Deptartment of Psychology, Ludwig-Maximilians-Universität München, Munich, Germany; 2 Department of Epileptology, University of Bonn, Bonn, Germany; 3 Institute for Cognitive Systems, Technische Universität München, Munich, Germany; 4 Graduate School of Systemic Neurosciences, LMU, Munich, Germany; 5 Birkbeck College, University of London, London, United Kingdom; University of Groningen, The Netherlands

## Abstract

Recalling information from visual short-term memory (VSTM) involves the same neural mechanisms as attending to an actually perceived scene. In particular, retrieval from VSTM has been associated with orienting of visual attention towards a location within a spatially-organized memory representation. However, an open question concerns whether spatial attention is also recruited during VSTM retrieval even when performing the task does not require access to spatial coordinates of items in the memorized scene. The present study combined a visual search task with a modified, delayed central probe protocol, together with EEG analysis, to answer this question. We found a temporal contralateral negativity (TCN) elicited by a *centrally* presented go-signal which was spatially uninformative and featurally unrelated to the search target and informed participants only about a response key that they had to press to indicate a prepared target-present vs. -absent decision. This lateralization during VSTM retrieval (TCN) provides strong evidence of a shift of attention towards the target location in the memory representation, which occurred despite the fact that the present task required no spatial (or featural) information from the search to be encoded, maintained, and retrieved to produce the correct response and that the go-signal did not itself specify any information relating to the location and defining feature of the target.

## Introduction

Focusing attention on an item of a remembered representation of a visual scene does not differ substantially from focusing attention on an item of an actually observed scene. Imagine you are visiting Venice and are asked to close your eyes and recall whether there is a particular café at the Piazza San Marco, in the center of which you are now standing (with eyes closed). You search through your mental representation in a similar way to how you would scan a photograph, a map, or an actual visual scene. This situation is analogous to tasks used in laboratory settings to study visual short-term memory (VSTM). One of the key questions in VSTM research is the degree to which selecting items from memory representation uses overlapping mechanisms with selecting items in perception. Recently, an increasing amount of evidence has been obtained supporting the view that encoding, maintenance, and recall from VSTM is associated with orienting of visuospatial attention towards a memorized representation that preserves the spatial layout of the encoded scene. Spatial attention has been found to be involved in VSTM maintenance [1]: attention was shifted towards the location where the VSTM-relevant item had previously been presented, which was indicative of the involvement of spatial attention in the updating of memorized information. Nobre et al. [2] used functional Magnetic Resonance Imaging (fMRI) to establish that areas associated with attentional orienting in visual search and orienting in the VSTM partially overlap. In particular, the authors reported areas in the posterior parietal cortex (PPC) to be involved in the orienting of attention to both visual and memory representations [2]. The hypothesis of overlapping networks was then extended from simple visual features to more complex perceptual representations (faces and scenes) [3]. The authors found that orienting attention to memorized representations of either faces or houses selectively modulated activity in fusiform or parahippocampal gyri, respectively. From this, it was concluded that object-based attention may be operating during the maintenance of relevant information in VSTM [3]. Spatially informative retro-cues (cues presented after a stimulus) have been found to influence the efficacy of VSTM recall [4], [5], in terms of both reaction times and accuracy of recall. This suggests that cueing attention towards a location within a memory representation influences recall and, consequently, that attentional orienting is involved in memory processes.

Further support for the hypothesized involvement of spatial attention in the maintenance and retrieval from spatially organized VSTM representations comes from new evidence obtained with the use of the so-called central probe paradigm and the ERP analyses of the EEG signal [6]–[10]. In the central probe paradigm, an event sequence on a trial starts with a memory array presented bilaterally, which is followed by a blank screen allowing for information maintenance. Then, a memory probe is presented centrally, which triggers recall from the VSTM. The results suggest that recalling a color or a shape from a certain location in VSTM elicits neural processes similar to attending to a location of an actually observed scene with a given spatial layout [6], [8]–[9]. In particular, an N2pc (see below) has been observed to be elicited by a non-lateralized visual memory probe. The N2pc is typically observed as a difference in mean amplitudes of the ERPs at posterior-occipital electrode sites contralateral vs. ipsilateral to an attended item in a visual display, around 180–300 ms after display onset [11]–[12]. Since the N2pc is thought to reflect the deployment of spatial attention to a location in a scene (e.g., [11]–[12], see also [13] for review), the N2pc elicited by non-lateralized probes in VSTM tasks has been interpreted as reflecting orienting of attention to a location within a memorized array with preserved spatial layout.

A more recent study [14] was designed to further specify the event-related lateralization (ERL) related to centrally presented retrieval cues (the component that was described as the memory-related N2pc [6], [8]–[10]). The authors observed that scalp topography associated with retrieval from the VSTM was more anterior as compared to the maintenance-related SPCN as well as the typical N2pc. The retrieval-related lateralization was most pronounced over the CP5/6 electrodes, while the N2pc and SPCN were observed over PO7/8 electrode pairs. The authors termed the retrieval-related lateralization *temporal contralateral negativity* (TCN), to distinguish it from the typical N2pc and maintenance-related SPCN. The distinguishable topographies of the TCN and SPCN components led the authors to suggest distinct processes involved in retrieval from memory representations and memory maintenance, even though the mechanisms might overlap to some degree.

### Aim of study

The aim of the present study was to establish whether focusing spatial attention on a memory representation that preserves spatial layout would occur even when the retrieval cue does not make any spatial reference. This question was motivated by the fact that to date, all studies examining spatial attention during retrieval processes used centrally presented cues that repeated one of the lateralized features (e.g., the color or shape of a lateralized item). Even though in some studies, such as [14] the repeated feature of the cue (color) was not directly response-relevant (participants were instructed to retrieve the orientation of a line element contained in a shape within the memorized search array that had the same color as the cue), the color was repeated. Accordingly, the observed retrieval-related lateralization might have resulted from repetition priming.

Moreover, none of the available studies (see review above) asked whether spatial attention is oriented to a location within a memory representation even when orienting is not expressly required by the task. Rather, in all studies, the cue made some reference – typically spatial – to the memory array, which itself had a lateralized spatial layout. Thus, for example, participants were asked to report whether the cue color had appeared in the earlier display [6], what color an item was at a position in the memory display indicated by a symbolic cue [10], or what orientation a bar had which was contained within a shape of the same color as the cue [14]. All these tasks refer participants to the memorized display and require selection of an item from this representation. In other words: focusing attention on a location or a localized item within the memory representation is implied by the task.

By contrast, the present study was designed to examine whether spatial attention would be allocated to a memory representation even if the task does not per se refer to the spatial layout of – and thus does not require participants to select a location within – the memory representation. To this end, we substituted the typically used retrieval cues (that make some form of spatial reference to the memorized display) by a stimulus that bore absolutely no spatial relation, and did not involve feature repetition with respect to the memorized display. In our paradigm, the initial visual search display (with lateralized layout) was followed by a maintenance interval, and participants were asked to respond to the presence/absence of a search target only upon presentation of a non-lateralized go-signal, without being told to encode the spatial location of the target. Importantly, the stimulus-response mapping was not known to participants prior to the presentation of the go-signal, so that they were unable to prepare the response during the maintenance interval.

We reasoned that if spatial attention is recruited during retrieval from the memory representation (although this is not explicitly required by the task), we should observe a lateralized activity related to the non-lateralized go-signal – which did not make any spatial or feature-based reference to the (lateralized) visual search display. Following [14] we reasoned that if the go-locked lateralization resembled the TCN pattern and had a differential topography to the search-locked N2pc/SPCN, this would indicate that the go-signal-related processes involved in retrieval are not simply a prolongation of memory maintenance (as reflected in the SPCN), but rather are indicative of a separate process of spatial-attentional orienting within a memory representation that preserves the display's spatial layout.

## Methods

### Participants

Fourteen paid volunteers (2 men, aged 22 – 28 years, *M* = 25.5 years) took part in the experiment, all with normal or corrected-to-normal vision. Two participants were excluded from analyses due to poor performance (>30% of errors). Mean error rate for the remaining participants was 12%, with a standard deviation of +/−3%.

### Stimuli and apparatus

**Figure 1:**
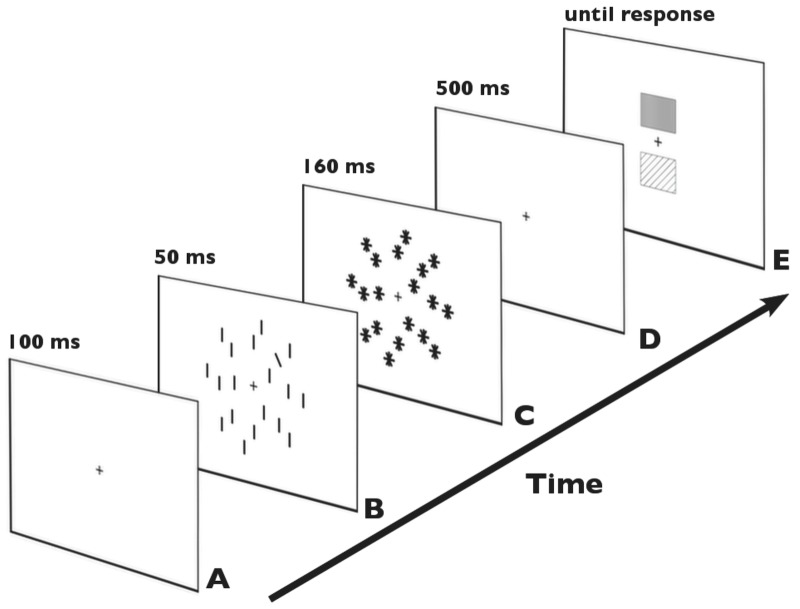
Example trial sequence. (A) fixation cross, (B) a target-present visual-search display, (C) post-display mask, (D) delay period, and (E) go-signal with red/green squares presented above/below the fixation cross in randomized order.

Stimuli were presented on a 17-inch computer screen with a 100-Hz refresh rate, placed at a distance of 100 cm from the observer. The visual search display consisted of black bars, on a white background, positioned on three imaginary circles with diameters of 5.1°, 7.4° and 9.1° of visual angle, respectively, around a central fixation cross (Figure 1B). There were two types of search displays: target-present (50%) and target absent (50%). Each target-present array contained 18 distractors (vertical bars, each 0.87°×0.13° in size) and one singleton target: a bar tilted 30° to the left from the vertical, of the same size and luminance as the distracters. The target could appear randomly at one of four positions on the intermediate circle: upper left/right or lower left/right relative to the center. The mask consisted of a display with 19 asterisks (produced by a combination of horizontal, vertical, 30° right-tilted, and 30° left-tilted bars). The go-signal consisted of two squares (1.2°×1.2°) positioned above and below the fixation cross (+/−0.6°); one of the squares was red (RGB: 255, 0, 0) and the other was green (RGB: 0, 128, 0). The upper/lower location of each of the colors was randomized across trials. Importantly, the go-signal was presented centrally and did not contain any lateralized information. The response keys were embedded in an international standard keyboard positioned under the observer's hands. Responses were executed using the key U (up, pressed with the right-hand index finger) or N (down, pressed with the left-hand index finger).

### Procedure

An experimental trial started with a fixation cross presented for 100 ms (see Figure 1A). Subsequently, a visual search display was presented for 50 ms (see Figure 1B) and immediately followed by a mask display presented for 160 ms; see Figure 1C. Participants were required to discern the presence (vs. absence) of an orientation singleton and maintain this information (‘target-present’ or ‘-absent’) during the subsequent delay period (500 ms, Figure 1D). Next, a go-signal was presented until response (see Figure 1E). Participants were instructed to respond to *target presence* with one of their index fingers pressing as fast as possible the key indicated by the green square, and to *target absence* by pressing the key indicated by the red square, as presented in the go-signal, which always consisted of both squares (green and red; see Figure 1). The mapping between the target stimulus and the color contained in the go-signal was constant (green for target present and red for target absent), as was the mapping between the location of the squares in the-go signal and a response key (upper square for upper key on the keyboard and lower square for the lower key). However, since the vertical location of green/red squares in the go-signal was randomized across trials (on some trials, red square could appear at the top and green at the bottom, and vice versa on other trials), the stimulus-response mapping (target-present vs. target-absent mapped to the upper key (right hand) vs. lower key (left hand)) was variable across trials. This was done to prevent any response biases on target-present trials or preparation of response prior to the onset of the go-signal. On trials with an incorrect response, there was an immediate feedback: the word *error* presented for 200 ms. The experiment consisted of 320 trials in total, split into 4 blocks, with short breaks in between. Only correct target-present trials were analyzed.

### EEG recording

EEG was recorded with Ag-AgCl electrodes from 64 electrode sites of an active electrode system (ActiCAP, Brain Products, GmbH, Munich, Germany). Horizontal and vertical EOG were recorded bipolar from the outer canthi of the eyes and from above and below the observer's left eye, respectively. All electrodes were referenced to Cz and re-referenced offline to the average of all electrodes. Electrode impedances were kept below 5 kΩ. Sampling rate was 500 Hz, with an online high cut-off filter of 125 Hz and an offline 30-Hz high cut-off filter (Butterworth zero phase, 24 dB/Oct).

### Data analysis: ERP data

#### Search-locked ERPs

The data was averaged over 900-ms epochs including a 200-ms pre-stimulus baseline, time-locked to search display onset. Segments with eye movements and eye blinks on any recording channel (indicated by any absolute voltage difference in a 200-ms segment exceeding 80 µV or voltage steps between two sampling points exceeding 50 µV) were excluded from analyses. Additionally, channels with other artifacts were separately excluded if amplitude exceeded +/−80 µV for a whole epoch or activity was lower than 0.10 µV for a 100-ms interval. Only correct trials were subjected to analyses.

In order to investigate the lateralized components, the EEG signal was epoched separately for left and right targets for the PO7/8 as well as the CP5/6 electrodes. The PO7/8 electrode pair was selected as posterior-occipital site representative for the N2pc/SPCN component, while the CP5/6 electrode pair was selected as temporal site representative for the TCN component. Since we were interested in examining whether the topography of the search-locked laterality is distinct from the laterality locked to the go-signal, we included both electrode pairs in the analyses of both the search-locked and the go-locked ERPs.

Mean amplitudes in the critical time windows (please see below) were calculated for the left and right target position conditions. For the main analysis, the mean amplitude values were averaged across left/right target conditions, resulting in 2 waveforms (contralateral vs. ipsilateral to the target of the visual search display) for each electrode pair. A 2×2 repeated-measures analysis of variance (ANOVA) with the factors laterality (contra- vs. ipsilateral) and electrode pair (PO7/8 vs. CP5/6) was performed on the mean amplitudes obtained for the PO7/8 and CP5/6 electrodes in the160–260 ms time window (standard N2pc window, +/−50 ms around the latency of the grand average peak of the difference between the contralateral and the ipsilateral electrode sites within the 150–250 time window); and in the 400–700-ms time window (retention interval prior to go-signal onset). To test whether there was an influence of target position (left vs. right) on the laterality effects in the search-locked N2pc time window (PO7/8 electrode pair) as well as in the go-signal locked TCN window (CP5/6 electrode pair), we conducted additional analyses with the factors target position (left vs. right) and laterality for the PO7/8 electrodes.

#### Go signal-locked ERPs

The data were averaged over 700 ms epochs including a 200-ms pre-stimulus baseline, time-locked to the onset of the go-signal. Artifact rejection procedures were identical as in the case of search-locked analyses. Also only correct trials were analyzed. Similarly to the search-locked ERPs, the EEG signal was epoched separately for left and right targets (of the display preceding the go-signal) for the PO7/8 and CP5/6 electrodes and then the left/right target conditions were averaged together, resulting in 2 waveforms for each electrode pair. A 2×2 repeated-measures analysis of variance (ANOVA) with the factors laterality (contra- vs. ipsilateral) and electrode pair (PO7/8 vs. CP5/6) was performed on the mean amplitudes obtained for PO7/8 and CP5/6 electrodes in the 80–120-ms time window relative to go-signal onset (+/-20 ms around the latency of the grand average peak of the difference between the contralateral and the ipsilateral electrode sites within the 80–120 ms time window), corresponding to the P1 component evoked by the go-signal; and finally on the mean amplitudes obtained for the PO7/8 and CP5/6 electrodes in the 180–300-ms time window, relative to go-signal onset, corresponding to the TCN component evoked by the go-signal.

#### Behavioral data

Statistical analyses were conducted on mean response times (RTs) and mean error rates. Prior to computing mean RTs, errors and outliers (trials with RTs exceeding +/−3SD from mean RT for each participant) were excluded. The remaining data (mean RTs and mean error rates) were submitted to one-way within-subject ANOVAs with factor trial type (target present vs. target absent).

## Results

### ERP Data

#### ERPs related to the search display

**Figure 2:**
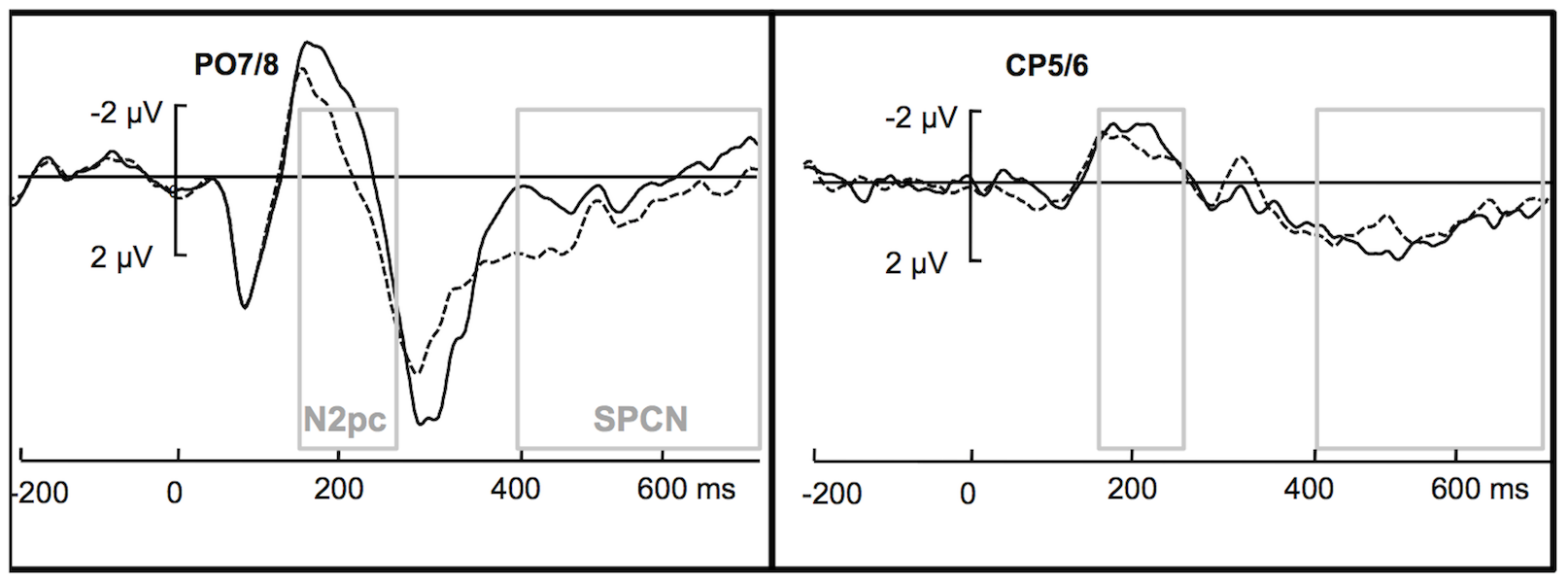
Display-locked ERPs. Grand Averages of the EEG signal for correct trials recorded from the PO7/8 electrode sites (left) and the CP5/6 sites (right) time-locked to search display onset, recorded contralaterally (dashed line) and ipsilaterally (solid line) to the target. The rectangular areas represent the analyzed time windows of the display-locked N2pc (160–260 ms) and the display-locked SPCN (400–700 ms). Where indicated by “N2pc” or “SPCN”, the laterality effect was significant. The search-locked waveforms were baseline-corrected relative to the 200-ms time window preceding display onset.

The analysis on mean EEG signal amplitude for the PO7/8 and CP5/6 electrodes in the standard N2pc time window (160–260 ms) locked to the onset of visual search display revealed a significant effect of laterality, *F*(1,11) = 15.052, *p* = .003, *η*_p_^2^ = .578, indicating a clear N2pc (see Figure 2), and an interaction between the two factors [laterality and electrode pair], *F*(1,11) = 27.657, *p*<.001, *η*_p_^2^ = .715. Separate comparisons revealed that the laterality effect was significant for the PO7/8 electrode pair (M_Contra_ = −1.48 µV, SEM = .5; M_Ipsi_ = −.32 µV, SEM = .5), *t*(11) = 4.924, *p*<.001 (two-tailed), but not for the CP5/6 electrode pair (M_Contra_ = 1.05 µV, SEM = .4; M_Ipsi_ = −.76 µV, SEM = .3), *t*(11) = 1.49, *p* = .164 (two-tailed). Note that the N2pc at the PO7/8 electrodes did not depend on the side of target presentation: when target side was examined as a factor, its interaction with laterality was not significant, *F*(1,11) = .903, *p* = .362, *η*_p_^2^ = .076.

In the later window (400–700 ms), while there was no overall (main) effect of laterality, *F*(1,11) = 2.401, *p* = .15, *η*_p_^2^ = .179, the electrode pair × laterality was significant, *F*(1,11) = 18.971, *p* = .001, *η*_p_^2^ = .633. Separate comparisons revealed that for the PO7/8 electrode pair, the laterality effect was significant (M_Contra_ = .219 µV, SEM = .6; M_Ipsi_ = 1.06 µV, SEM = .5), *t*(11) = 2.841, *p* = .016 (two-tailed), suggestive of an SPCN effect (see Figure 2), while no such effect was evident for the CP5/6 electrode pair, (M_Contra_ = 1.09 µV, SEM = .2; M_Ipsi_ = .91 µV, SEM = .3), *t*(11) = 1.07, *p* = .306 (two-tailed).

#### ERPs related to the go-signal

**Figure 3:**
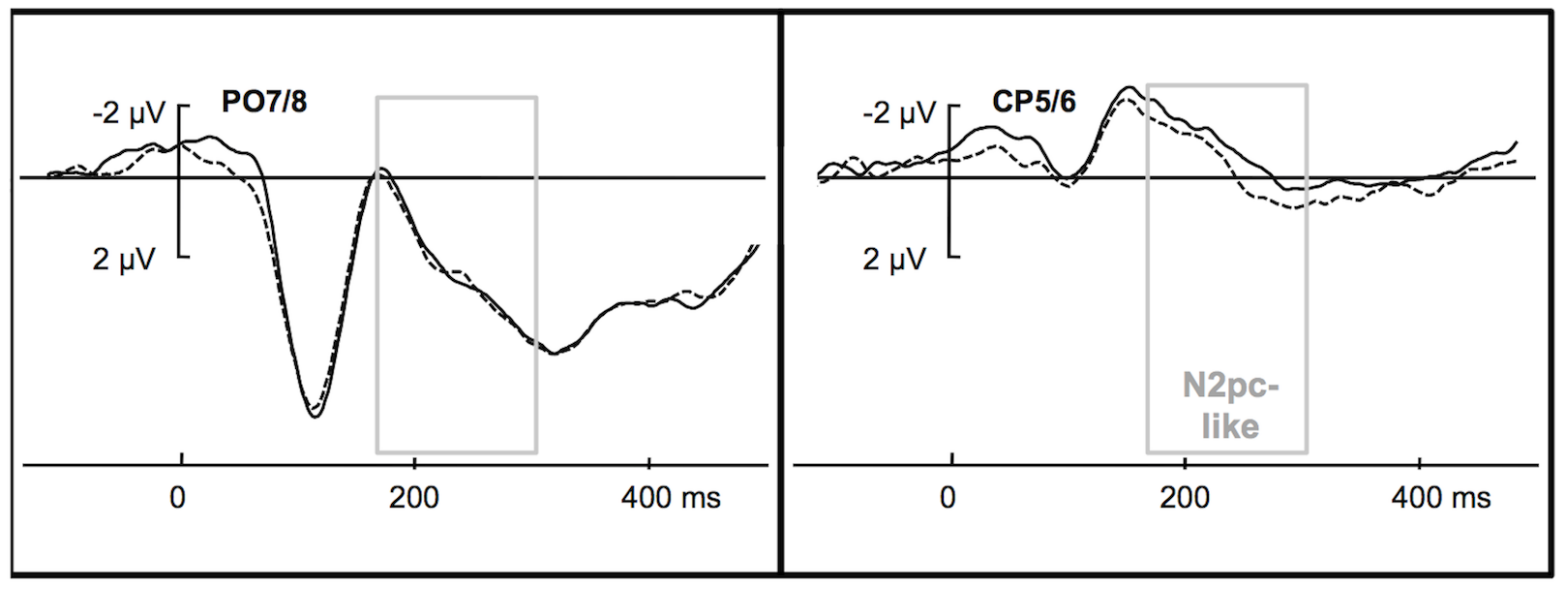
Go-signal locked ERPs. Grand Averages of the EEG signal for correct trials recorded from the PO7/8 electrode sites (left) and the CP5/6 sites (right) time-locked to go-signal onset recorded contralaterally (dashed line) or ipsilaterally (solid line) to the target that presented in the preceding visual-search display. The rectangular area represents the analyzed time windows of the go-signal-locked TCN (180–300 ms). Where indicated by “TCN”, the laterality effect was significant. The go-signal-locked waveforms were baseline-corrected relative to the 200-ms time window preceding go-signal onset.

The analysis on the mean EEG signal amplitudes in the 80–120-ms time window relative to go-signal onset revealed no overall effect of laterality, *F*(1,11) = .024, *p* = .881, *η*_p_^2^ = .002; although the interaction was significant, *F*(1,11) = 5.391,*p* = .04, *η*_p_^2^ = .324, a significant laterality effect could not be ascertained for any of the electrode pairs: *t*(11) = .845, *p* = .416 (two-tailed) for the PO7/8 electrodes (M_Contra_ = 5.78 µV, SEM = .8; M_Ipsi_ = 5.63 µV, SEM = .8); and *t*(11) = 1.233, *p* = .243 (two-tailed) for the CP5/6 electrodes (M_Contra_ = −.226 µV, SEM = .2; M_Ipsi_ = −0.36 µV, SEM = .2), suggesting that the sensory P1 component related to the go-signal was not associated with any lateralized processing, see Figure 3. Analysis of the subsequent TCN effect in the time window 180–300 ms post go-signal onset indicated a marginally significant laterality × electrode interaction, *F*(1,11) = 4.074, *p* = .069, *η*_p_^2^ = .27. Separate comparisons revealed that in this time window, in contrast to the search-locked effects, the laterality effect was significant for the CP5/6 electrode pair (M_Contra_ = −.77 µV, SEM = .4; M_Ipsi_ = −.35 µV, SEM = .3), *t*(11) = 2.46, *p* = .032 (two-tailed), but not for the PO7/8 electrode pair (M_Contra_ = 3.45 µV, SEM = .1; M_Ipsi_ = 3.51 µV, SEM = 1), *t*(11) = −.246, *p* = .81 (two-tailed). Note that when the analysis was performed on the neighboring pair of electrodes (i.e., the PO3/4), the laterality × electrode interaction turned out significant, *F*(1,11) = 5.84, *p* = .034, *η*_p_^2^ = .35. The laterality effect was also not significant for the PO3/4 electrode pair, *t*(11) = 1.41, *p* = .187 (two-tailed), indicative of a dissociation relative to the CP5/6 electrode pair (similarly to the dissociation between the PO7/8 and CP5/6 electrode pairs). Furthermore, the CP5/6 electrodes did not depend on the side of target presentation either: when target side was included as a factor, its interaction with laterality was not significant, *F*(1,11) = 2.278, *p* = .159, *η*_p_^2^ = .172.

#### Correlational analyses

**Figure 4:**
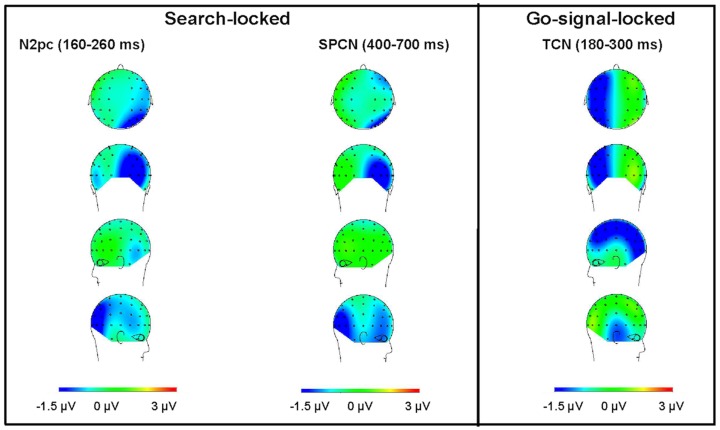
Scalp topography of the laterality effects. Topographies calculated as the mean voltage contralateral minus ipsilateral, for all lateral electrodes pairs, separately for the three time windows of analysis: the display-locked N2pc (160–260 ms, relative to visual-search display onset), left panel; the display-locked SPCN (400–700 ms, relative to visual-search display onset), middle panel; and the go-signal-locked TCN (180–300 ms, relative to go-signal onset), right panel. The maps in each panel represent the view from the top, from the back, and the two lateral views, respectively. Warm colors represent positive voltages, cold colors negative voltages.

The scalp topography of the go-locked TCN component differed from the topography related to the display-locked SPCN and, respectively, the N2pc, see Figure 4. The dissociation in the laterality effects between the CP5/6 and PO7/8 electrode pairs in the various windows analyzed confirm that the TCN is a different ERP component to the SPCN/N2pc components. Nevertheless, in order to provide corroborative evidence that the go-locked TCN is not a simple prolongation of the SPCN, but rather a distinct component, we examined whether there is a significant Pearson correlation between the laterality effects in the respective two time windows. Mean amplitude values on the ipsilateral side were subtracted from mean amplitude values on the contralateral side for each participant separately, resulting in one “laterality effect” value for each participant, separately for each time window. The results were as follows: The SPCN effect measured at the PO7/8 electrodes (400–700 ms, relative to search onset) did not significantly correlate with the laterality effect measured at the CP5/6 electrodes (180–300 ms, relative to go-signal onset), *r* (10) = −.437, *p* = .156. Also, the laterality effect obtained at the CP5/6 electrodes in the time window of the SPCN did not correlate with the laterality effect obtained in the time window of 180–300 ms relative to the go-signal, *r* (10) = −.21, *p* = .511.

### Behavioral data

A one-way ANOVA with trial type as factor (target-present vs. target-absent) revealed RTs to be faster on trials on which a target was present (M = 399 ms, SEM = 18) as compared to target-absent trials (M = 421 ms, SEM = 19), *F* (1,11) = 8.804; *p* = .013, *η*_p_^2^ = .44, consistent with the search literature, e.g., [15]–[17]. The error ANOVA did not yield a significant effect of trial type *F*(1,11) = 1.903, *p* = .195, *η*_p_^2^ = .15, though participants tended to respond conservatively (rather than liberally), as indicated by a greater rate of more miss (*M* = 11.33%) than false-alarm errors (*M* = 8.75%).

## Discussion

The aim of the present study was to investigate whether spatial attention is deployed to locations in a VSTM representation of a search array even though maintaining the spatial layout of the array was not required by the task. To examine this, we used a delayed-response visual-search task with a non-lateralized ‘go’-signal that itself did not convey any spatial information and did not make any spatial (or featural) reference to the memorized array. Rather, participants were only asked to discern the presence (vs. absence) of a target in the search array and indicate their decision in response to the delayed go-signal, without being required to encode or maintain any information about the target's location or defining feature during the delay. To answer the questions at issue, we examined the ERPs locked to the search display and, respectively, to the go-signal, focusing on the search-locked N2pc/SPCN and the go-locked TCN. Specifically, we asked whether a spatially (and featurally) non-informative, display-unrelated go-signal would elicit an event-related lateralization in the form of a TCN, and whether this lateralization would have a distinct topography relative to the N2pc/SPCN. We reasoned that finding such as TCN lateralization related to the go-signal would reflect the involvement of a separable ERP component, indicating that the VSTM representation mediating the required response does have a spatial format, even though spatial information was not necessary at all for performing the task. Also, since the memory probe (i.e., go-signal) did not share any feature with the display, the present paradigm avoided confounding of retrieval from VSTM with response priming by the retrieval probe. Consequently, finding a lateralization related to go-signal presentation would provide strong evidence that the memory representation underlying performance is truly spatial in nature.

In line with the predictions, we found that a go-stimulus, which did not make any spatial reference to the (memorized) search array and did not repeat any of its features indeed elicited a lateralized TCN. Importantly, the topography of the TCN was distinct from – namely, more anterior to – the topography of the search-locked N2pc/SPCN, as evidenced by the differential voltage distribution maps (see Figure 4) and significant interactions between electrode site and laterality effect in all three time windows of analysis (i.e., the N2pc, SPCN, and TCN): For the N2pc and SPCN time windows, the laterality effect was significant for the PO3/4 electrode pair but not manifest for the CP5/6 pair, this pattern was reversed for the TCN time window; furthermore, no significant correlation was observed between the magnitude of the SPCN and that of the TCN effect.

Following [14], we take our set of results to provide strong evidence that the observed TCN is a distinct component from the maintenance-related SPCN. The TCN observed in the present study exhibited a more anterior distribution to relative to the N2pc and SPCN (see Figure 4), in line with previous reports of the same component [14]. Also in line with previous reports [14], its polarity was characterized by a more negative deflection over sites contralateral (vs. ipsilateral) to the location of the memorized target. However, in the present study, TCN was elicited earlier: around 200 ms after the centrally presented go-signal – compared to [14], where the TCN peaked around 300 ms after the onset of the central cue. This difference in peak latency might be due to a difference in the stimuli used in the two studies. We used very simple stimuli, namely, vertical lines, with the target being defined as tilted singleton line. In contrast, rather complex compound stimuli were used in [14], with participants being required to retrieve two features – namely, the color of a circular item (target-defining feature) and the orientation of the line inside the circle (response-defining feature) – in response to a color cue that informed participants of the probed circle (to which they had to give a line-orientation response). Moreover, in [14] the target cued by the retrieval probe was not the only singleton in the memory array – in contrast to the target in the present paradigm. This might also have affected the latency of the TCN component.

The SPCN effect observed during the interval between the search array and the go-signal most likely reflects the maintenance of memorized contents, in line with standard interpretations of the SPCN [18]–[20]. By contrast, the TCN effect observed subsequent to go-signal presentation is likely to reflect spatial-attentional orienting to a location within memory representation associated with retrieval processes. That is, the go-signal most likely elicited active retrieval processes from a representation stored in a spatial format, as participants were asked to produce the search task response (target present vs. absent) upon this signal, after an interval of mere maintenance. This idea, of the involvement of active retrieval from VSTM, is corroborated by the relatively long RTs taken to produce a motor reaction to what is essentially a ‘prepared’ decision. Arguably, as part of the retrieval operation, the go-signal elicited orientation of spatial attention to a location in a memory representation in a rather automatic manner, as the go-signal used in the present paradigm provided information only about which response key to press to indicate the prepared decision, without necessitating any form of *active* (spatial or featural) memory search. In support of this, recall that the go-signal was presented *centrally*, that is, should not by itself have produced any lateralization of the EEG signal, as corroborated by the non-lateralized go-signal locked P1. Also, the go-signal did not make any spatial reference to the laterally organized search array and it did not repeat any target feature present in the search display, excluding any possibility of repetition priming. Despite these facts, a lateralized TCN effect was observed – strongly arguing in favor of spatial-attentional orienting within a memory representation even when this is not required or induced by the task. Finally, note that the TCN effect was observed even with the visual-search display being masked, rendering it unlikely that the memorized array was maintained in iconic memory. Yet, by virtue of its (apparent) spatiotopic organization, VSTM resembles iconic memory representations.

Taken together, the present findings confirm and extend previous research [3]–[4], [6], [8]–[10], [14] by showing that a stimulus which activates retrieval processes without referring in any way (spatially or featurally) to a memorized display engages processes of orienting of spatial attention to a location within a memory representation with preserved spatial layout. Hence, the TCN effect can be truly interpreted as reflecting post-sensory operations of spatial attention over a VSTM representation recruited during memory retrieval, even when encoding and/or retrieving spatial layout is not required by the task.
